# A Rare Case of Epidural Myeloma Presenting as Recurrent Subdural Bleeding

**DOI:** 10.7759/cureus.17794

**Published:** 2021-09-07

**Authors:** Keerthana P Sivakolundu, Aviraag Vijaya Prakash, Natasha M Savage, Vamsi K Kota, Kristina Zarkua

**Affiliations:** 1 Department of General Medicine, Government Kilpauk Medical College, Chennai, IND; 2 Department of Hematology and Oncology, Georgia Cancer Center at Augusta University, Augusta, USA; 3 Department of Pathology, Medical College of Georgia at Augusta University, Augusta, USA

**Keywords:** cns involvement, epidural myeloma, subdural hematoma, mri, multiple myeloma

## Abstract

Multiple myeloma (MM) is an incurable clonal B-cell malignancy that usually presents with neoplastic monoclonal plasma cells in either bone or soft tissues. Central nervous system involvement of the myeloma (CNS-MM), such as dural myeloma or intraparenchymal infiltration, or diffuse leptomeningeal involvement, is uncommon. Dural involvement of myeloma without parenchymal or leptomeningeal disease is even rarer, with only seven cases reported previously.

We present a case of epidural myeloma in a 50-year-old man with known kappa light chain MM, presenting with multiple episodes of subdural hemorrhage and progressive neurological deficits. He initially presented with severe back pain, hypercalcemia, and acute kidney injury (AKI). Further evaluation showed lytic bone lesions and elevated kappa light chains, and bone marrow biopsy showed 32% of clonal plasma cells. He was initially treated with bortezomib, lenalidomide, and dexamethasone combination, followed by pomalidomide and daratumumab. Eventually, he developed two episodes of subdural hemorrhage and left-sided seventh cranial nerve palsy, which was treated conservatively and monitored by computed tomography (CT) of the head. However, he gradually developed multiple cranial nerve palsies, weakness, and urinary incontinence. Cerebrospinal fluid (CSF) analysis showed elevated protein without any aberrant immunophenotype. Magnetic resonance imaging (MRI) of the brain showed diffuse smooth dural enhancement with extensive calvarial and skull base marrow replacement; MRI of the spine showed diffuse epidural enhancement in thoracic and lumbar regions, findings consistent with epidural myeloma. The patient received three doses of cranial irradiation but, unfortunately, could not tolerate further treatment and opted for hospice care.

Intracranial hemorrhage is common in MM patients, and it is important to consider CNS involvement in patients presenting with recurrent subdural hemorrhage and to perform imaging (preferably MRI) earlier in the disease course. Due to its rarity, the treatment of CNS-MM is very heterogeneous. Thus, case reporting is important to accumulate data on this rare presentation.

## Introduction

Multiple myeloma (MM) is an incurable malignant neoplasm of plasma cells that account for approximately one percent of all cancers and 10% of hematologic malignancies in the United States [1]. The malignant plasma cells are generally confined to the bone marrow and the vascular compartment; however, dissemination can occur through the bone or blood, resulting in extramedullary disease. The extramedullary involvement occurs only in three to five percent of patients and usually involves the sinuses, nasopharynx, oropharynx, gastrointestinal tract, and lungs [2].

Neurological symptoms in MM most commonly occur due to complications such as spinal cord compressions by bone lesions, paraprotein-related neuropathy, hypercalcemia, hyperviscosity, or amyloidosis [3], while neurological symptoms due to CNS involvement of MM itself are rare and occur in one percent of patients [4]. The CNS involvement can manifest as a solitary cerebral lesion, intraparenchymal infiltration, diffuse leptomeningeal disease, or isolated dural involvement, while isolated dura mater involvement is extremely rare. The common symptoms of intracranial myeloma include extremity weakness, altered mental status, and cranial nerve palsies, and the prognosis is poor with a median overall survival rate of 1.5 to three months from the time of diagnosis of CNS-MM [4].

## Case presentation

A 50-year-old male was referred to our care from a private facility after being diagnosed with stage I (according to International Staging System [ISS]) kappa light chain MM.

In April 2020, he presented with severely worsening non-traumatic left-sided upper back pain, radiating to the midline and down to his mid-lower back. Further evaluation showed multiple lytic lesions in bones - largest at T6 and mild compression deformities at T8 and T10 along with hypercalcemia (13.5 mg/dl), AKI (creatinine: 1.7 mg/dl, blood urea nitrogen: 34.1, and glomerular filtration rate: 45.3 ml/min), and mild anemia. He also had a right lower lobe pulmonary embolus without evidence of right ventricular strain at that time. He was hospitalized and was treated with intravenous hydration, zoledronic acid, and apixaban. He was started on prephase treatment with 40 mg dexamethasone intravenously for six days for suspected MM.

**Figure 1:**
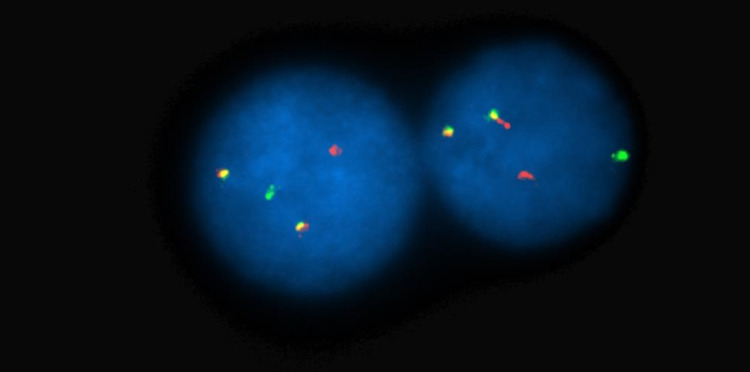
FISH with probes targeted to plasma cell myeloma revealing t(11;14); BCL1 (11q13) in green fused to IGH (14q32) in red resulting in yellow fusion signals FISH, Fluorescence in situ hybridization; IGH, immunoglobulin heavy chain gene.

Further laboratory results are as follows: Urine 24-hour protein excretion was increased with monoclonal protein present in urine protein electrophoresis. Serum protein electrophoresis showed no monoclonal protein; however, the kappa/lambda light chain panel showed an increased immunoglobulin kappa light chains of 1378 mg/dl and an increased kappa/lambda ratio (K:L) of 233.59. Beta-2 microglobulin levels were also increased at 3.0 mg/dl, albumin was normal at 3.5 mg/dl, and lactate dehydrogenase (LDH) levels were normal. The immunoglobulin (Ig) levels were low (IgG: 627 mg/dl, IgA: 25.6 mg/dl, and IgM < 26 mg/dl). Peripheral blood smear showed normocytic anemia and bone marrow aspirate, and biopsy confirmed the presence of 32% of clonal plasma cell population with cytoplasmic kappa light chain restriction. Fluorescence in situ hybridization (FISH) showed TP53 gene deletion at 17p13.1 and translocation (11,14) resulting in the fusion of CCND1 (BCL1) at 11q13 with immunoglobulin heavy chain gene (IGH) at 14q32 (Figure 1).

On presentation, his Eastern Cooperative Oncology Group (ECOG) performance status was one, and Karnofsky performance score was 90%. Treatment was initiated with bortezomib, lenalidomide, and dexamethasone along with allopurinol and valacyclovir for infectious prophylaxis. The patient’s pain was managed with as-needed narcotics. The treatment was well tolerated without any major complications other than mild neuropathy that was treated with gabapentin. After completing four cycles of chemotherapy, he was evaluated for autologous stem cell transplant and consented for stem cell mobilization and collection. However, mobilization was held because he tested positive for severe acute respiratory syndrome coronavirus 2 (SARS-COV-2). After recovery from the COVID-19 infection, he was diagnosed with depression and anxiety with suicidal ideation, for which he received treatment with antidepressants. It was decided to hold on to the transplant transiently until the patient recovers.

**Figure 2:**
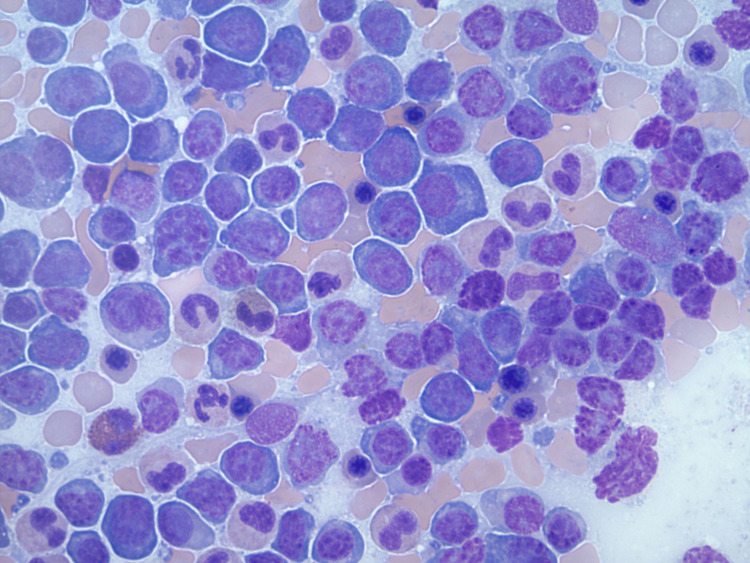
Bone marrow aspirate smears revealing decreased trilineage hematopoiesis with an increase in plasma cells including many with lymphoplasmacytic morphology Lymphoglandular bodies are abundant in the background (Wright-Giemsa, 500x).

In January 2021, his K:L increased to 67.47, and he was initiated on lenalidomide and dexamethasone considering his disease progression. After treatment, his K:L transiently decreased to 40.69; however, it increased to 99.64 in April 2021. Repeat bone marrow biopsy showed 80% plasma cell proliferation (Figure 2), and FISH detected t(11,14); a gain of 1q and chromosome 5; and loss of chromosome 13, p53, and 5q. He was started on pomalidomide and subcutaneous daratumumab in anticipation of a bone marrow transplant in the future. Shortly after, he developed left-sided Bell's palsy with no other neurological deficits. CT of the head revealed subdural hematoma possibly secondary to thrombocytopenia, which was managed conservatively with serial head CTs. Apixaban and pomalidomide were discontinued, and he received platelet transfusions to maintain a platelet count of >50,000 cells/mm^3^.

Several months later, he was admitted to the hospital with double vision and slurring of speech. On clinical examination, he was alert and oriented; cranial nerve examination showed left-sided Bell’s palsy along with left sixth nerve palsy and right-sided tongue deviation with no sensory or motor deficits. CT brain without contrast showed right anterior para alpine subdural hematoma measuring approximately 2 mm in thickness, which is unchanged from prior along with chronic left holohemispheric subdural hematoma with areas of new acute intracranial hemorrhage, consistent with acute on chronic subdural hematoma. He could not complete MRI because of his severe back pain. He required no acute intervention, and he received packed red blood cell (RBC) and platelet transfusions for pancytopenia to keep platelets > 100,000 cells/mm^3^ and hemoglobin > 7 g/dl.

**Figure 3:**
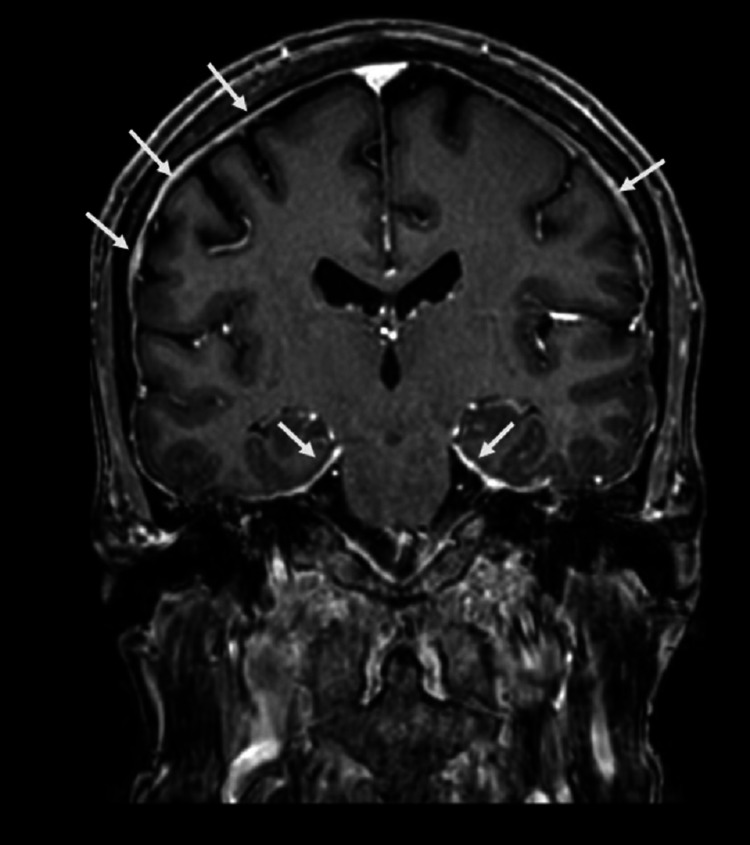
MRI of the brain postcontrast T1 showing diffuse dural enhancement

A week later, he was hospitalized again for hyponatremia and ground-level fall. He also complained of dysphagia and difficulty opening his right eyelid. Complete neurological examination revealed bilateral multiple cranial nerve palsies, involving the third nerve on the right, minimal eye movement in any direction on the left, bilateral seventh nerve, and tongue deviation to the right along with mild (-4/5) weakness in the right upper and lower extremities. He also developed progressive weakness and urinary retention during hospitalization. His K:L was > 500; however, his beta-2 microglobulin, albumin, and LDH levels were normal. CT of the brain showed scattered calvarial lucencies, which is compatible with the known diagnosis of MM. CSF analysis showed increased protein of 1088 mg/dl, five white blood cells (WBCs), and 230 RBCs, and flow cytometry did not show any aberrant immunophenotype. MRI of the brain was done after pain control, which revealed poorly enhancing soft tissue infiltrating the cavernous sinuses bilaterally and diffuse smooth dural enhancement with extensive calvarial and skull base marrow replacement along with left subacute to chronic frontoparietal subdural hematoma (Figure 3). MRI spine revealed diffuse epidural enhancement of the thoracic and lumbar spinal segments, more pronounced at T9-T10 and L5-S1. The neuro-oncology team reviewed the MRI and stated that the clinical and MRI findings are consistent with diffuse epidural involvement of MM. The patient received three cycles of cranial radiation. However, he could not tolerate the whole course of treatment due to severe back pain and claustrophobia. He opted for hospice and palliative care.

## Discussion

CNS-MM is a rare form of the extramedullary disease characterized by plasma cell infiltration of the CNS, meninges, or CSF [5]. It is usually seen as extramedullary relapses in about two to three years after the initial diagnosis of MM, but it is also observed at the time of diagnosis in some patients [6]. The median age of CNS-MM diagnosis is often younger (50- to 60-year-old group) when compared to the usual median age of diagnosis of MM. However, the age at presentation varied between studies [5]. CNS-MM can arise in any stage of the disease [7], although an association between CNS-MM and the later stage of the disease was found in a previous study [8].

Several risk factors have been identified for the development of CNS-MM. Deletions of chromosome 17p13.1 (p53) have been found in 89% of the CNS-MM patients [3]. In a retrospective study by Jurczyszyn et al., 39% of patients with CNS-MM had del(13q) and 23% had del(17p) [7]. Elevated LDH, lack of CD56 expression, an adhesion molecule of plasma cells, and plasmablastic morphology have also been shown to be associated with increased risk of CNS-MM [7,9]. In our patient, deletion of 17p (p53) may be the potential risk factor for the CNS spread, and CD56 expression was not tested. However, Egan et al. noted no significant association between cytogenetics, cytology, and histopathology at diagnosis and subsequent development of CNS-MM [5].

Intracranial myeloma can be classified into four groups as follows: (a) those extending from the skull growing inward, (b) those growing from the dura mater or the leptomeninges, (c) intraparenchymal lesions, and (d) those arising from the mucous membrane of a nasopharyngeal plasmacytoma. Among these groups, leptomeningeal involvement is the most common form [10]. Isolated dural involvement without spreading from the bone and parenchymal or meningeal spread is a very rare scenario [11]. There are only eight cases of isolated extraosseous epidural myeloma reported in the literature [12]. Leptomeningeal involvement by myeloma is often associated with CSF paraproteinemia and/or CSF plasmacytosis [13]. Though CSF cytology has a false negative rate of 10%-15% [14], the absence of CSF plasma cells and negative flow cytometry in our patient leans toward the fact that the myeloma involvement was limited to the dura mater, sparing the arachnoid.

CNS myeloma can cause heterogeneous neurological symptoms related to either spinal, cranial, or meningeal disease [8]. The symptoms are not exclusive to CNS infiltration by MM and can be found in other disease-related situations such as hypercalcemia, uremia, hyperviscosity syndrome, as well as side effects of drug therapy, and, in some cases, amyloid protein [3]. One such confounding factor that should be considered in myeloma patients is intracranial bleeding. MM patients are at increased risk for bleeding due to many factors such as interference of myeloma-produced antibodies against clotting factors or amyloid damage of the endothelium and platelet dysfunction [15]. Our patient had recurrent episodes of subdural hemorrhage, and the multiple cranial nerve palsies were initially thought to be secondary to the acute on chronic subdural hematoma.

CNS-MM has a wide variety of imaging manifestations, ranging from lesions with an extra-axial, a focal or diffuse meningeal enhancement to solid or diffuse intra-axial, intraparenchymal nodules or masses [15]. Contrast-enhanced MRI is more sensitive than CT and constitutes the method of choice in the detection of CNS-MM [8]. This is consistent with our patient’s imaging results, where the CT scan failed to detect the signs of epidural involvement. MRI showed diffuse epidural dural enhancement involving the cavernous sinus and the thoracic and lumbar spine, explaining the third, sixth, and seventh cranial nerve palsies, weakness, and urinary retention, respectively. Previous studies have shown a false negative rate of 10% with MRI [8]. Therefore, it is appropriate to perform imaging, pathological, and CSF examinations concurrently. However, in daily clinical practice, it is not always possible to perform all three tests due to poor performance status, end-stage disease, or patient refusal [7].

The optimal approach for the treatment of CNS-MM is not well studied because of the relatively small number of patients presenting with this complication. Various multimodal treatment options have been proposed, including cranial radiotherapy, intrathecal chemotherapy, and systemic chemotherapy [13]. Cranial radiation was reported in one review to show statistically significant benefit in the median survival rate compared to those not receiving this treatment [8]. The drugs that are shown to be efficacious in CNS-MM are bortezomib [16], thalidomide and lenalidomide [17], pomalidomide (immunomodulator class), and marizomib (a second-generation proteasome inhibitor) [18,19]. Two studies have also shown longer survival rates in patients who received intrathecal therapy with drugs such as methotrexate, steroids, and cytarabine [17,20].

The survival rate in CNS-MM varies between studies. According to Jurczyszyn et al., those receiving MM therapy before CNS involvement and having more than one cytogenetic marker of poor prognosis were risk factors that reduced the median survival rate from 25 months to 5.5 months when either was present and to two months when both were present [8]. Our patient received extensive chemotherapy and immunomodulator therapy before CNS-MM and has more than one cytogenetic marker of poor prognosis, thus indicating a median prognosis of about two months according to the study [8].

## Conclusions

Isolated epidural myeloma, without leptomeningeal or brain parenchymal involvement, is extremely rare. It usually presents with non-specific neurological symptoms including multiple cranial nerve palsies, which can be confounded by various myeloma-related disease conditions such as intracranial bleeding. The multiple episodes of subdural hematoma were initially thought to contribute to the neurological symptoms in our patient, delaying the diagnosis of CNS-MM. Thus, it is important to consider CNS involvement as a differential diagnosis in MM patients presenting with recurrent subdural hematoma.

A variety of treatment strategies for CNS-MM have been adopted, but the prognosis remains grim, except in rare case reports. We treated our patient with three cycles of cranial irradiation. However, he was not able to complete treatment due to intolerance, and so we will not be able to assess the effect of this treatment modality in epidural myeloma.
